# Crystal structure of 4-meth­oxy­quinazoline

**DOI:** 10.1107/S1600536814025082

**Published:** 2014-11-21

**Authors:** Gamal A. El-Hiti, Keith Smith, Amany S. Hegazy, Mohammed B. Alshammari, Benson M. Kariuki

**Affiliations:** a Cornea Research Chair, Department of Optometry, College of Applied Medical Sciences, King Saud University, PO Box 10219, Riyadh 11433, Saudi Arabia; b School of Chemistry, Cardiff University, Main Building, Park Place, Cardiff CF10 3AT, Wales; c Chemistry Department, College of Sciences and Humanities, Salman bin Abdulaziz University, PO Box 83, Al-Kharij 11942, Saudi Arabia

**Keywords:** crystal structure, 4-meth­oxy­quinazoline, quinazoline derivatives, π–π stacks, herringbone packing

## Abstract

The title compound, C_9_H_8_N_2_O, is almost planar, with the C atom of the meth­oxy group deviating from the mean plane of the quinazoline ring system (r.m.s. deviation = 0.011 Å) by 0.068 (4) Å. In the crystal, mol­ecules form π–π stacks parallel to the *b*-axis direction [centroid–centroid separation = 3.5140 (18) Å], leading to a herringbone packing arrangement.

## Related literature   

For the synthesis of quinazoline derivatives, see: Bogert & May (1909 ▶); Smith *et al.* (2005 ▶); Wang *et al.* (2010 ▶); Yang *et al.* (2010 ▶); Han *et al.* (2012 ▶). For the crystal structures of related compounds, see Alshammari *et al.* (2014 ▶); Derabli *et al.* (2013 ▶); Gao *et al.* (2012 ▶); Huang & Tan (2012 ▶); Jia *et al.* (2011 ▶).
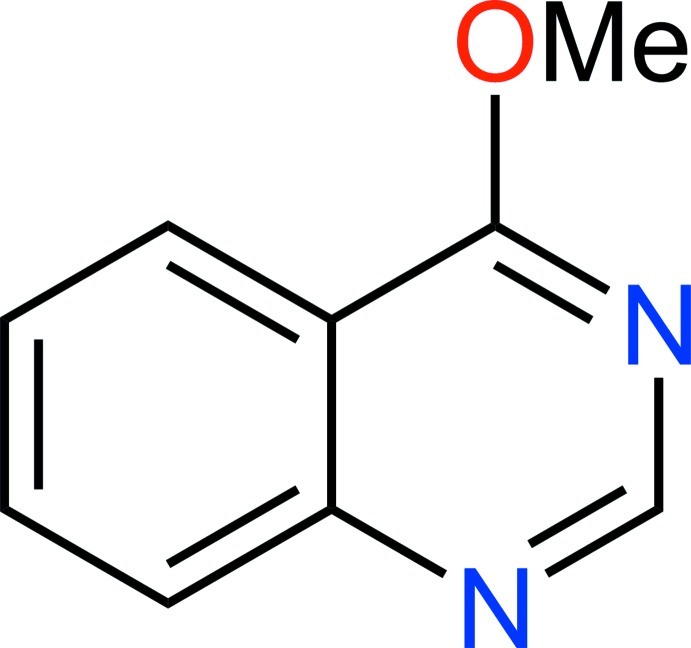


## Experimental   

### Crystal data  

C_9_H_8_N_2_O
*M* *_r_* = 160.17
Monoclinic, 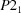

*a* = 6.9590 (6) Å
*b* = 4.0517 (3) Å
*c* = 13.5858 (12) Å
β = 91.754 (8)°
*V* = 382.88 (6) Å^3^
*Z* = 2
Cu *K*α radiation
μ = 0.77 mm^−1^
*T* = 150 K
0.57 × 0.12 × 0.08 mm

### Data collection  

Agilent SuperNova (Dual, Cu at zero, Atlas) diffractometer
Absorption correction: gaussian (*CrysAlis PRO*; Agilent, 2014 ▶) *T*_min_ = 0.641, *T*_max_ = 0.895
2166 measured reflections
1435 independent reflections
1311 reflections with *I* > 2σ(*I*)
*R*_int_ = 0.040

### Refinement  

*R*[*F* ^2^ > 2σ(*F* ^2^)] = 0.054
*wR*(*F* ^2^) = 0.159
*S* = 1.08
1435 reflections
110 parameters
1 restraint
H-atom parameters constrained
Δρ_max_ = 0.24 e Å^−3^
Δρ_min_ = −0.23 e Å^−3^

### 

Data collection: *CrysAlis PRO* (Agilent, 2014 ▶); cell refinement: *CrysAlis PRO*; data reduction: *CrysAlis PRO*; program(s) used to solve structure: *SHELXS2013* (Sheldrick, 2008 ▶); program(s) used to refine structure: *SHELXL2013* (Sheldrick, 2008 ▶); molecular graphics: *ORTEP-3 for Windows* (Farrugia, 2012 ▶); software used to prepare material for publication: *WinGX* (Farrugia, 2012 ▶) and *CHEMDRAW Ultra* (Cambridge Soft, 2001 ▶).

## Supplementary Material

Crystal structure: contains datablock(s) I, New_Global_Publ_Block. DOI: 10.1107/S1600536814025082/hb7317sup1.cif

Structure factors: contains datablock(s) I. DOI: 10.1107/S1600536814025082/hb7317Isup2.hkl

Supporting information file. DOI: 10.1107/S1600536814025082/hb7317Isup3.cml

**Fig. 1.**
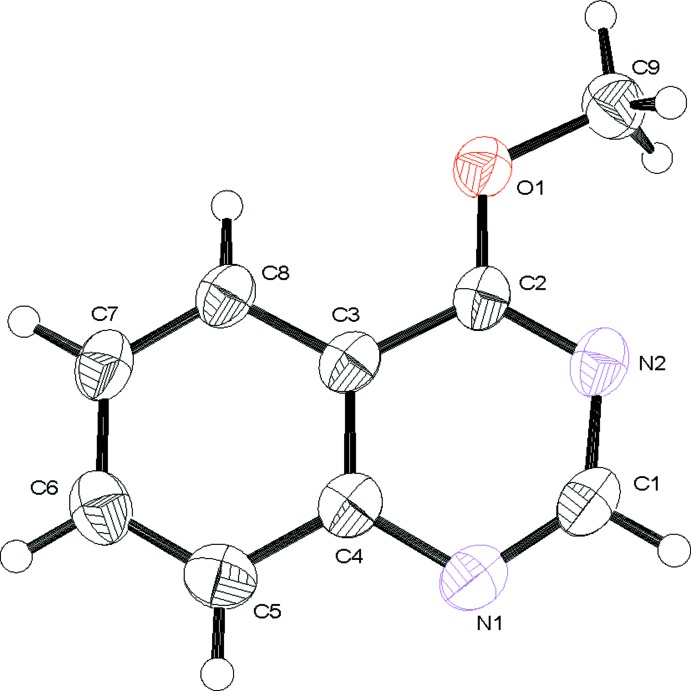
The title molecule showing 50% probability displacement ellipsoids.

**Fig. 2.**
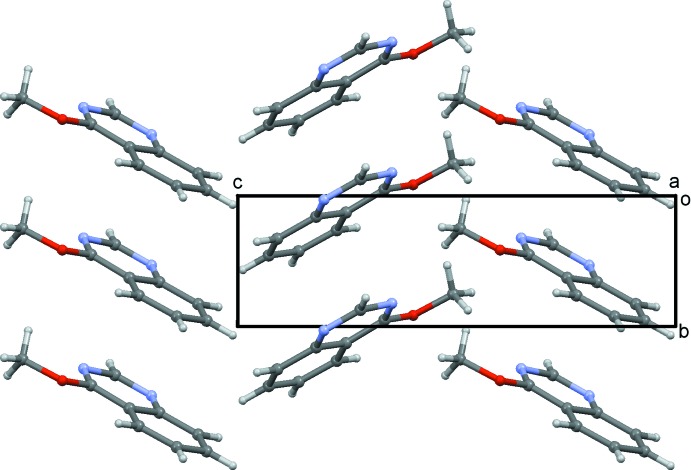
Crystal packing viewed down the a axis.

. DOI: 10.1107/S1600536814025082/hb7317fig1.tif
The title mol­ecule showing 50% probability displacement ellipsoids.

a . DOI: 10.1107/S1600536814025082/hb7317fig2.tif
Crystal packing viewed down the *a* axis.

CCDC reference: 1034363

Additional supporting information:  crystallographic information; 3D view; checkCIF report

## supplementary crystallographic information

### S1. Structural commentary

Copper-catalyzed processes offer convenient approaches to various quinazoline derivatives starting from (2-bromo­phenyl)­methyl­amines, 2-amino­benzyl­amines or 2-bromo­benzo­nitriles (Wang *et al.*, 2010; Yang *et al.*, 2010; Han *et al.*, 2012). For ring substitution and modification of 4-meth­oxy­quinazolines, see: Smith *et al.* (2005). 4-Meth­oxy­quinazoline was synthesized in 81% yield from reaction of quinazoline-4(3H)-thione with iodo­methane in aqueous methanol containing potassium hydroxide at room temperature for 24 h (Bogert & May, 1909). For the X-ray structures for related compounds, see Alshammari *et al.* (2014); Derabli *et al.* (2013); Gao *et al.* (2012); Huang & Tan (2012); Jia *et al.* (2011).

The asymmetric unit consists of one molecule of C_9_H_8_N_2_O (Fig. 1). The molecule is almost planar apart from the methyl hydrogen atoms, with C9 deviating from the least squares plane of the quinazoline group by 0.068 (4)Å. The molecules form π-π stacks parallel to the *b*-axis leading to a herring-bone pattern in the crystal structure (Fig. 2).

### S2. Synthesis and crystallization

To a solution of quinazoline-4(3*H*)-thione (4.9 g, 30.2 mmol) in a 1:1 mixture of methanol and water (50 ml) containing potassium hydroxide (3.0 g), was added iodo­methane (5.7 g, 40.1 mmol) at room temperature. The reaction mixture was stirred for 24 h, then methanol was removed under reduced pressure and the remaining aqueous layer was extracted with di­ethyl ether (2 × 20 ml). The organic layer was separated, washed with water (2 × 10 ml), dried (MgSO_4_), and evaporated under reduced pressure. The residue obtained was purified by column chromatography (silica gel, di­ethyl ether–hexane, 1:1) to give 4-meth­oxy­quinazoline (3.9 g, 24.4 mmol, 81%) as a white solid. Crystallization from a mixture of ethyl acetate and di­ethyl ether (1:3 by volume) gave the title compound as colorless needles. mp 34–35°C [lit. 35.4°C; Bogert & May, (1909)]. ^1^H NMR (400 MHz, CDCl_3_, δ p.p.m.): 8.77 (s, 1 H, H-2), 8.08 (dd, *J* = 1, 8 Hz, 1 H, H-8), 7.88 (dd, *J* = 1,8 Hz, 1 H, H-5), 7.76 (app. dt, *J* = 1, 8 Hz, 1 H, H-7), 7.50 (app. t, *J* = 8 Hz, 1 H, H-6), 4.13 (s, 3 H, OCH_3_). ^13^C NMR (100 MHz, CDCl_3_, δ, p.p.m.): 167.4 (*s*, C-4), 154.6 (*d*, C-2), 151.1 (*s*, C-8a), 133.8 (*d*, C-7), 128.0 (*d*, C-8), 127.3 (*d*, C-5), 123.8 (*d*, C-6), 116.9 (*s*, C-4a), 54.6 (*q*, OCH_3_). EI–MS (*m/z*, %): 160 (*M*^+^, 68), 131 (32), 130 (30), 103 (100), 90 (22), 76 (28), 63 (21). CI–MS (*m/z*, %): 161 (*MH*^+^, 100). HRMS (CI): calculated for C_9_H_9_N_2_O [*MH*] 161.0709; found, 161.0708.

### S3. Refinement

Crystal data, data collection and structure refinement details are summarized in Table 1.

### Crystal data

**Table:**

C_9_H_8_N_2_O	*F*(000) = 168
*M**_r_* = 160.17	*D*_x_ = 1.389 Mg m^−^^3^
Monoclinic, *P*2_1_	Cu *K*α radiation, λ = 1.54184 Å
*a* = 6.9590 (6) Å	Cell parameters from 1311 reflections
*b* = 4.0517 (3) Å	θ = 3.3–67.7°
*c* = 13.5858 (12) Å	µ = 0.77 mm^−^^1^
β = 91.754 (8)°	*T* = 150 K
*V* = 382.88 (6) Å^3^	Needle, colourless
*Z* = 2	0.57 × 0.12 × 0.08 mm

### Data collection

**Table:**

Agilent SuperNova (Dual, Cu at zero, Atlas) diffractometer	1311 reflections with *I* > 2σ(*I*)
ω scans	*R*_int_ = 0.040
Absorption correction: gaussian (*CrysAlis PRO*; Agilent, 2014)	θ_max_ = 74.6°, θ_min_ = 3.3°
*T*_min_ = 0.641, *T*_max_ = 0.895	*h* = −8→8
2166 measured reflections	*k* = −4→5
1435 independent reflections	*l* = −11→16

### Refinement

**Table:**

Refinement on *F*^2^	1 restraint
Least-squares matrix: full	Hydrogen site location: inferred from neighbouring sites
*R*[*F*^2^ > 2σ(*F*^2^)] = 0.054	H-atom parameters constrained
*wR*(*F*^2^) = 0.159	*w* = 1/[σ^2^(*F*_o_^2^) + (0.110*P*)^2^ + 0.0483*P*] where *P* = (*F*_o_^2^ + 2*F*_c_^2^)/3
*S* = 1.08	(Δ/σ)_max_ < 0.001
1435 reflections	Δρ_max_ = 0.24 e Å^−^^3^
110 parameters	Δρ_min_ = −0.23 e Å^−^^3^

### Special details

**Table:**

Experimental. Absorption correction: CrysAlisPro, Agilent Technologies, Version 1.171.37.33 (release 27-03-2014 CrysAlis171 .NET) (compiled Mar 27 2014,17:12:48) Numerical absorption correction based on gaussian integration over a multifaceted crystal model Empirical absorption correction using spherical harmonics, implemented in SCALE3 ABSPACK scaling algorithm.
Geometry. All e.s.d.'s (except the e.s.d. in the dihedral angle between two l.s. planes) are estimated using the full covariance matrix. The cell e.s.d.'s are taken into account individually in the estimation of e.s.d.'s in distances, angles and torsion angles; correlations between e.s.d.'s in cell parameters are only used when they are defined by crystal symmetry. An approximate (isotropic) treatment of cell e.s.d.'s is used for estimating e.s.d.'s involving l.s. planes.

### Fractional atomic coordinates and isotropic or equivalent isotropic displacement parameters (Å^2^)

**Table:**

	*x*	*y*	*z*	*U*_iso_*/*U*_eq_	
C1	−0.1179 (5)	0.8736 (9)	0.7198 (2)	0.0403 (8)	
H1	−0.2391	0.7831	0.7078	0.048*	
C2	0.1829 (4)	0.9515 (7)	0.6650 (2)	0.0330 (7)	
C3	0.2338 (4)	1.1283 (7)	0.7527 (2)	0.0330 (7)	
C4	0.0855 (4)	1.1608 (7)	0.8208 (2)	0.0349 (7)	
C5	0.1265 (5)	1.3290 (8)	0.9096 (3)	0.0426 (8)	
H5	0.0308	1.3526	0.9554	0.051*	
C6	0.3034 (5)	1.4570 (8)	0.9292 (2)	0.0431 (8)	
H6	0.3276	1.5675	0.9883	0.052*	
C7	0.4520 (5)	1.4246 (10)	0.8608 (3)	0.0411 (7)	
H7	0.5731	1.5127	0.8751	0.049*	
C8	0.4169 (5)	1.2640 (7)	0.7739 (2)	0.0364 (7)	
H8	0.5138	1.2440	0.7287	0.044*	
C9	0.2674 (5)	0.7530 (9)	0.5079 (3)	0.0433 (8)	
H9A	0.1669	0.8757	0.4745	0.065*	
H9B	0.2226	0.5340	0.5215	0.065*	
H9C	0.3771	0.7410	0.4669	0.065*	
N1	−0.0945 (4)	1.0312 (7)	0.8028 (2)	0.0412 (7)	
N2	0.0121 (4)	0.8237 (6)	0.6479 (2)	0.0378 (7)	
O1	0.3208 (3)	0.9159 (6)	0.59930 (16)	0.0381 (6)	

### Atomic displacement parameters (Å^2^)

**Table:**

	*U*^11^	*U*^22^	*U*^33^	*U*^12^	*U*^13^	*U*^23^
C1	0.0293 (13)	0.0351 (16)	0.0562 (19)	−0.0019 (13)	−0.0033 (12)	0.0037 (15)
C2	0.0334 (14)	0.0278 (15)	0.0374 (15)	0.0007 (13)	−0.0059 (11)	0.0054 (12)
C3	0.0310 (14)	0.0268 (14)	0.0408 (15)	0.0051 (11)	−0.0036 (12)	0.0073 (12)
C4	0.0356 (16)	0.0283 (15)	0.0405 (16)	0.0033 (13)	−0.0020 (12)	0.0071 (13)
C5	0.0473 (18)	0.0386 (17)	0.0423 (18)	0.0053 (15)	0.0052 (14)	0.0023 (15)
C6	0.0528 (19)	0.0364 (18)	0.0396 (17)	0.0030 (15)	−0.0075 (14)	−0.0008 (14)
C7	0.0367 (14)	0.0373 (16)	0.0486 (17)	0.0006 (14)	−0.0104 (12)	0.0020 (14)
C8	0.0327 (14)	0.0346 (16)	0.0416 (18)	0.0033 (13)	−0.0051 (12)	0.0057 (14)
C9	0.0445 (17)	0.0437 (18)	0.0414 (17)	0.0051 (16)	−0.0052 (13)	−0.0020 (14)
N1	0.0336 (13)	0.0372 (14)	0.0528 (17)	−0.0001 (11)	0.0023 (11)	0.0013 (13)
N2	0.0346 (13)	0.0336 (14)	0.0446 (14)	−0.0001 (10)	−0.0084 (11)	0.0044 (11)
O1	0.0346 (10)	0.0430 (13)	0.0367 (11)	−0.0004 (11)	−0.0016 (8)	−0.0004 (10)

### Geometric parameters (Å, º)

**Table:**

C1—N1	1.302 (4)	C5—H5	0.9300
C1—N2	1.366 (4)	C6—C7	1.417 (5)
C1—H1	0.9300	C6—H6	0.9300
C2—N2	1.311 (4)	C7—C8	1.363 (5)
C2—O1	1.338 (4)	C7—H7	0.9300
C2—C3	1.425 (4)	C8—H8	0.9300
C3—C8	1.409 (4)	C9—O1	1.445 (4)
C3—C4	1.413 (4)	C9—H9A	0.9600
C4—N1	1.373 (4)	C9—H9B	0.9600
C4—C5	1.407 (5)	C9—H9C	0.9600
C5—C6	1.355 (5)		
			
N1—C1—N2	128.6 (3)	C7—C6—H6	119.6
N1—C1—H1	115.7	C8—C7—C6	119.8 (3)
N2—C1—H1	115.7	C8—C7—H7	120.1
N2—C2—O1	120.3 (3)	C6—C7—H7	120.1
N2—C2—C3	123.2 (3)	C7—C8—C3	120.0 (3)
O1—C2—C3	116.5 (2)	C7—C8—H8	120.0
C8—C3—C4	120.2 (3)	C3—C8—H8	120.0
C8—C3—C2	124.6 (3)	O1—C9—H9A	109.5
C4—C3—C2	115.2 (3)	O1—C9—H9B	109.5
N1—C4—C5	119.8 (3)	H9A—C9—H9B	109.5
N1—C4—C3	121.9 (3)	O1—C9—H9C	109.5
C5—C4—C3	118.2 (3)	H9A—C9—H9C	109.5
C6—C5—C4	120.9 (3)	H9B—C9—H9C	109.5
C6—C5—H5	119.6	C1—N1—C4	115.5 (3)
C4—C5—H5	119.6	C2—N2—C1	115.6 (3)
C5—C6—C7	120.8 (3)	C2—O1—C9	116.9 (2)
C5—C6—H6	119.6		
