# Supplementary figures and images for: Crystal structure of 4-meth­oxy­quinazoline
Source: Acta Crystallogr Sect E Struct Rep Online. 2014 Nov 21;70(Pt 12):o1279. doi: 10.1107/S1600536814025082 (PMC4257374; doi:10.1107/S1600536814025082)

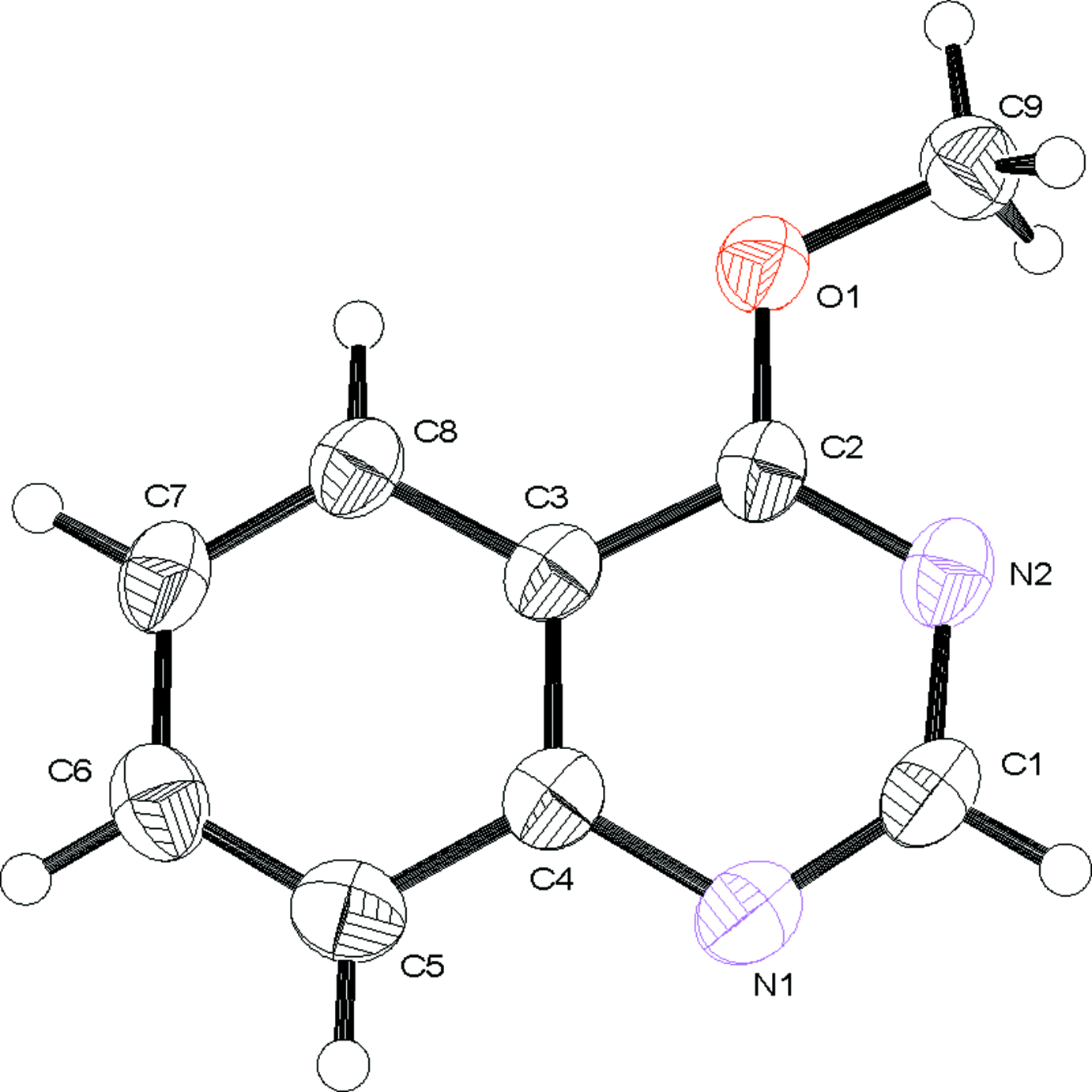

Supplement: Supplementary file 4 [file e-70-o1279-fig1.tif]

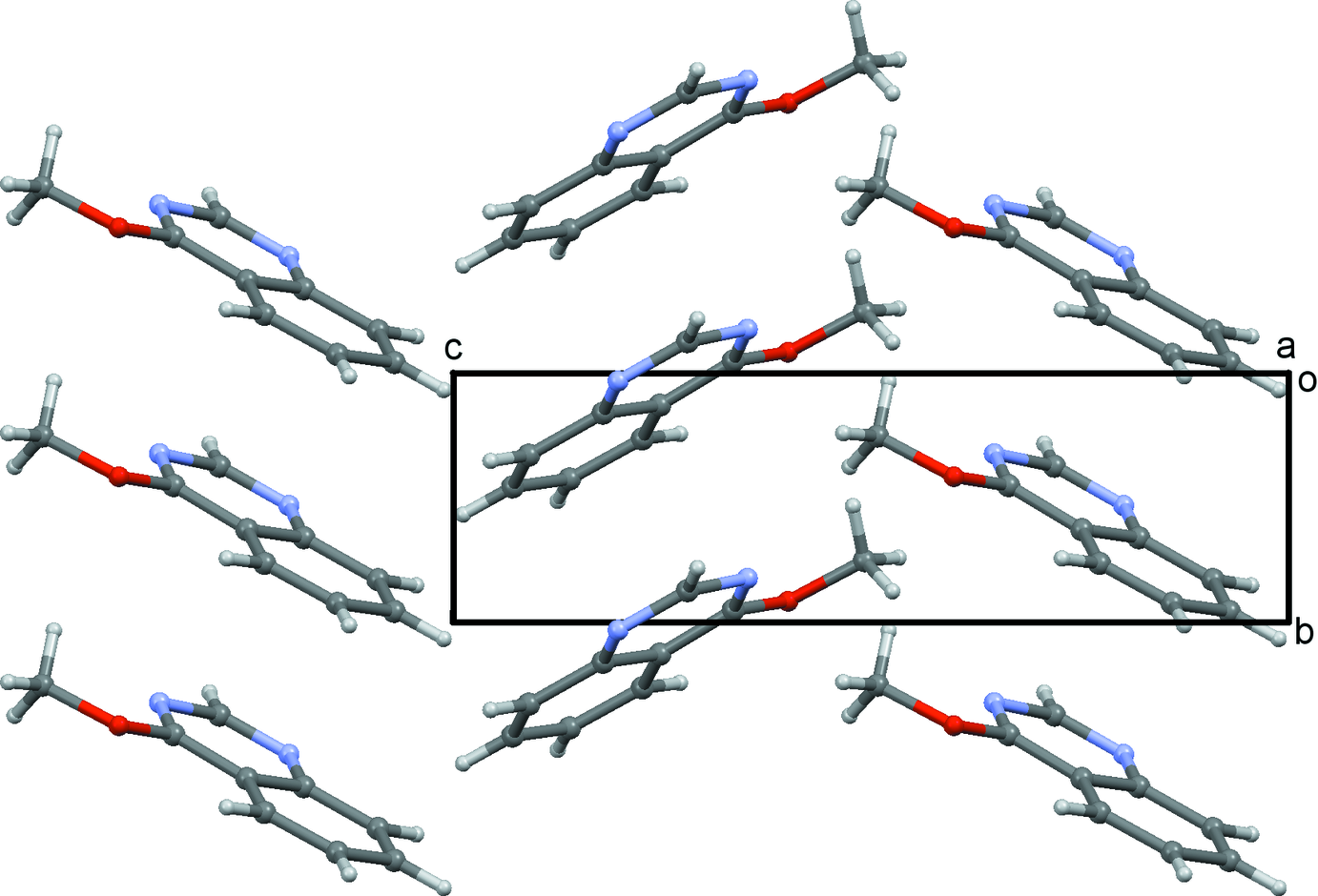

Supplement: Supplementary file 5 [file e-70-o1279-fig2.tif]
